# PLO genomic diversity underpins differential immunogenicity of *Trueperella pyogenes* strains from deer and swine

**DOI:** 10.3389/fvets.2026.1758657

**Published:** 2026-03-11

**Authors:** Huanyi Zhu, Xi Wang, Tenglong Zhao, Dong Tang, Yixi Sun, Xiaowei Yang, Guangwei Zhao

**Affiliations:** 1 College of Veterinary Medicine, Southwest University, Chongqing, China; 2 MOE Key Laboratory of Gene Function and Regulation, State Key Laboratory of Biocontrol and Guangdong Provincial Key Laboratory of Aquatic Economic Animals, School of Life Sciences, Sun Yat-Sen University, Guangzhou, China; 3 Zhenhe Tongchuang (Chongqing) High-Tech Technology Co., Ltd., Chongqing, China

**Keywords:** immunogenicity, pyolysin, *Trueperella pyogenes*, vaccine, virulence gene

## Abstract

*Trueperella pyogenes* (*T. pyogenes*), an opportunistic pathogen, colonizes animal mucosal membranes (respiratory, genitourinary, gastrointestinal) and globally causes systemic infections including endometritis and pneumonia. Its primary virulence factor, pyolysin (PLO), has been extensively studied to elucidate the pathogen’s mechanisms and to develop vaccines, yet effective prevention strategies have not been achieved. This study characterized two *T. pyogenes* strains isolated from sika deer (D-*T. pyogenes*) and swine (S-*T. pyogenes*) through comparative genomics and immunological analyses. Whole-genome sequencing revealed significant genetic divergence in the *plo* gene, with 85 nucleotide differences (94.7% identity) and 19 amino acid substitutions (96.4% identity) between the strains. The S-*T. pyogenes* strain possessed unique virulence factors, including nutritional toxicity and specialized secretion systems, which may explain its enhanced virulence in murine models. Immunization with prokaryotically expressed recombinant PLO proteins (D-rPLO and S-rPLO) elicited robust humoral and cellular immune responses in mice. While D-rPLO induced faster antibody production and higher survival rates post-challenge, S-rPLO triggered stronger pro-inflammatory cytokine responses (IL-2, TNF-*α*) but conferred inferior protection, likely due to immune evasion associated with its virulence-related epitopes. Flow cytometry analysis revealed a predominant increase in the CD4^+^/CD8^+^ T cell ratio, highlighting Th1-mediated immunity as critical for pathogen clearance. Histopathological examination correlated D-rPLO’s superior efficacy with reduced tissue damage, suggesting that host-specific variations in the *plo* gene influence antigenic epitope recognition. These findings highlight host-driven adaptations shaping bacterial immunogenicity and PLO’s functional diversity, advancing *T. pyogenes* pathogenesis understanding and guiding multi-antigen vaccine design targeting conserved epitopes for balanced efficacy.

## 1 Introduction

*Trueperella pyogenes* (*T. pyogenes*) is an opportunistic bacterium that infects a variety of animals, including cattle, sheep, and pigs. It causes diseases such as mastitis, abscesses, endometritis and pneumonia (1–4), leading to significant economic losses. Antibiotics like *β*-lactams, tetracyclines, and macrolides are commonly used to treat *T. pyogenes* infections (5–7). However, the rise of antimicrobial resistance due to excessive antibiotic use has become an increasing concern. Since effective preventive strategies are currently lacking, developing vaccines is essential for controlling this disease.

*T. pyogenes*, is a gram-positive, pleomorphic, short rod-shaped bacterium that lacks capsules, spores, and flagella (8). It carries several virulence factors, including Nan, CbpA, and Fim, which help the bacterium adhere to and colonize host cells (9–11). Among these, pyolysin (PLO) is especially important in disease development. PLO is a 57.9 kDa pore-forming toxin found in all *T. pyogenes* strains isolated so far (12), encoded by the *plo* gene within a 1,605-base pair open reading frame. It attaches to host cell membranes and creates transmembrane pores that compromise membrane integrity (13). This cytolytic effect enables *T. pyogenes* to destroy red blood cells and cause death in neutrophils, macrophages, and epithelial cells (14, 15). Importantly, PLO demonstrated strong immunogenicity capable of eliciting robust immune responses (16), making it a promising candidate for vaccine development against *T. pyogenes* infections.

Of the various strategies developed against *T. pyogenes*, several PLO-targeting vaccines have shown promise. Formaldehyde-inactivated recombinant PLO offers protective efficacy (17), while chimeric antigens provide partial protection (18, 19). DNA vaccines have been particularly well-studied: for instance, pVAX1-PLO co-immunized with IL-1β enhances immune activation and protection (20). Moreover, CpG ODN-containing DNA vaccines designed from multi-epitopes of *plo*, *cbpA*, *fimA*, and *nanH* elicit robust humoral and cellular immunity (21). Heterologous prime-boost regimens combining DNA and subunit vaccines further strengthen immune responses (22).

Epidemiological data show that *T. pyogenes* populations often harbour multiple co-circulating strains (23), with single-strain infections being rare—suggesting ongoing host-imposed selection. Genomic comparisons confirm that diversity lies mainly within the accessory genome (24). In contrast, the *plo* gene is highly conserved across strains, especially in functional domains such as those conferring hemolytic activity (25). Despite high genetic variability among isolates, PLO remains notably conserved compared to other virulence factors, supporting its suitability for broad-coverage vaccines.

In this study, we isolated and characterized two *T.pyogenes* strains from deer and swine and employed whole-genome sequencing to systematically compare their virulence factors and genomic structures. Furthermore, we assessed the immunogenicity and vaccine potential of the PLO proteins, providing valuable insights to support the development of recombinant subunit vaccines targeting this emerging pathogen.

## 2 Materials and methods

### 2.1 Animal care and ethics statement

The Kunming mice utilized in this study were obtained from the Chongqing National Bio-industry Base Experimental Animal Center. Kunming mice used in this study were euthanized by a physical method of cervical dislocation, in accordance with the AVMA Guidelines for the Euthanasia of Animals (*2020 Edition*). All experimental methods and animal welfare guidelines received approval from the Institutional Animal Care and Use Committee (IACUC) of Southwest University (approval number IACUC-20250611-01).

### 2.2 Bacterial isolation and purification

A strain was isolated from the lung tissue of an 18-month-old sika deer that died on a farm in Yongchuan, Chongqing Municipality. Before death, the deer showed symptoms including depression, loss of appetite, and purulent nasal discharge. A postmortem examination revealed widespread lung lesions with hemorrhagic and necrotic changes. Another strain was obtained from the lung tissue of a sick pig on a farm in Luzhou, Sichuan Province. The pig exhibited signs of depression, fever, and lung abscesses. Samples were cultured on 5% rabbit blood agar and incubated at 37 °C in anaerobic conditions with 5% CO_2_ for 48 h. After incubation, distinct colonies were isolated, subcultured, and purified for further analysis.

### 2.3 Bacterial identification and characterization

Gram staining was performed, and bacterial morphology was examined using an Olympus optical microscope at 1000× magnification. Purified colonies were inoculated into 5 mL tryptic soy broth (TSB) supplemented with 5% fetal bovine serum (FBS) and incubated at 37 °C with shaking (180 rpm) for 18 h. Bacterial cells were harvested by centrifugation (4,000× *g*, 4 °C), washed twice with phosphate-buffered saline (PBS), and subjected to negative staining with 2% (w/v) phosphotungstic acid prior to transmission electron microscopy (TEM) imaging.

Genomic DNA was extracted from bacterial isolates using a standardized protocol. The 16S rRNA gene was amplified by PCR using universal primers (Table 1) following established methodologies (26, 27). Amplicons were sequenced, and the resulting data were analyzed using the NCBI BLAST platform for preliminary identification.

**Table 1:** Primers used in this study.

Primer	Sequence (5′–3′)
16S-F	AGAGTTTGATCCTGGCTCAG
16S-R	ACGGCTACCTTGTTACGACTT
D-*T. pyogenes*-F	CCGGAATTCGCCGGATTGGGAAACAGCT
D-*T. pyogenes*-R	CCGCTCGAGAGGGCTTGACGTTTTCCTCGAC
S-*T. pyogenes*-F	CCGGAATTCATGAAACGAAAGGCTT
S-*T. pyogenes*-R	CCGCTCGAGTGGATTTGACATTGTCC
β-actin-F	CCACTGTCGAGTCGCGTCC
β-actin-R	ATTCCCACCATCACACCCTGG
IL-2-F	CCCACTTCAAGCTCCACTTC
IL-2-R	ATCCTGGGGAGTTTCAGGTT
IL-10-F	CTTACTGACTGGCATGAGGATCA
IL-10-R	GCAGCTCTAGGAGCATGTGG
TNF-α-F	ACGGCATGGATCTCAAAGAC
TNF-α-R	GTGGGTGAGGAGCACGTAGT

### 2.4 Genomic sequencing and analysis

Genomic DNA was extracted from bacterial isolates and its purity was measured using a NanoDrop spectrophotometer, with OD_260/280_ values between 1.8 and 2.0 and OD_260/230_ values between 2.0 and 2.2. The DNA was sheared to an average fragment size of 350 base pairs using a Covaris M220 sonicator, followed by library construction using the Illumina TruSeq™ DNA PCR-Free Kit. Paired-end sequencing (2 × 150 bp) was carried out on an Illumina HiSeq 2,500 system.

Raw sequencing reads were demultiplexed with CASAVA (version 1.8.2) and quality filtered using Trimmomatic (version 0.38) with parameters set to trim bases in a 4-base sliding window if their Phred quality score was below 3, ensuring that reads shorter than 100 bp after trimming were discarded. The filtered reads were assembled *de novo* using SPAdes genome assembler (version 3.12.0) with default settings. Assembly quality was evaluated using QUAST (version 5.0.2) to assess contig statistics, and CheckM (version 1.1.3) to determine genome completeness and contamination levels.

Virulence genes were identified by querying the Virulence Factor Database (VFDB), The *E*-value threshold for annotation was set to 1e-6. Multiple sequence alignments of nucleotide and protein sequences were performed with ClustalX 2.0 and visualized using Jalview. B-cell epitope prediction was conducted using the BepiPred-2.0. Secondary structure analysis of the PLO protein was performed using the SOPMA online tool, and three-dimensional structural modeling was generated via the Phyre2 web server. The variant residues (highlighted in yellow) were mapped onto the predicted tertiary structure using ChimeraX, and their spatial distribution was visualized based on residue surface accessibility. Surface accessibility of amino acid residues was represented using different parameter values, with thresholds set at 0.1 (blue), 0.4 (white), and 0.7 (red).

### 2.5 Pathogenicity assay

A total of 78 mice were randomly assigned to 13 groups (*n* = 6 per group), with Group 13 designated as the negative control. For the isolate derived from deer, bacterial suspensions were serially diluted in sterile saline to obtain the desired concentrations. Concurrently, forty-two mice were distributed into seven groups (n = 6 per group) for the swine-derived isolate. Based on preliminary studies, the deer-derived suspensions were prepared at 8.63 × 10^11^, 6.86 × 10^11^, 5.49 × 10^11^, 4.03 × 10^11^, 2.86 × 10^11^, and 1.68 × 10^11^ concentrations, while the swine-derived suspensions were adjusted to concentrations of 3.00 × 10^8^, 2.25 × 10^8^, 1.50 × 10^8^, 1.00 × 10^8^, 8.75 × 10^7^, and 7.50 × 10^7^ colony-forming units per milliliter (CFU/mL). All mice were administered intraperitoneal injections of either the bacterial suspensions or sterile saline in the case of the negative control group. Mortality was recorded daily over a seven-day period following inoculation, and the median lethal dose (LD_50_) was calculated using the modified Karber method (28).

### 2.6 Expression of recombinant PLO protein

Utilizing the sequencing-derived coding sequence of the *plo* gene, restriction sites for *EcoR*I and *Xho*I, along with corresponding primers, were designed employing SnapGene 6.0 and Primer Premier 5.0 software (Table 1). Following polymerase chain reaction (PCR) amplification and verification, the *plo* gene was inserted into the pET32a plasmid vector. Positive bacterial colonies were identified through PCR screening, and the integrity of the recombinant plasmids was further confirmed by restriction enzyme digestion with *EcoR*I and *Xho*I, as well as by sequencing performed by BGI Genomics. The recombinant plasmid pET32a-PLO was subsequently transformed into *E.coli* BL21 (DE3) cells. Expression of the recombinant PLO protein (rPLO) was induced using isopropyl *β*-D-thiogalactoside (IPTG). Following induction, bacterial cells were lysed via ultrasonication at 4 °C, and the expression of rPLO was validated through sodium dodecyl sulfate-polyacrylamide gel electrophoresis (SDS-PAGE) and Western blot analysis employing anti-His tag antibodies. Critical parameters influencing protein expression, including induction duration (ranging from 1 to 6 h), temperature (16 °C to 37 °C), and IPTG concentration (0.1 to 1.0 mM), were systematically optimized. The recombinant protein, initially denatured in 8 M urea, was refolded through gradient dialysis and subsequently utilized for downstream functional assays.

### 2.7 Animal experiments and sample collection

**Figure 1:**
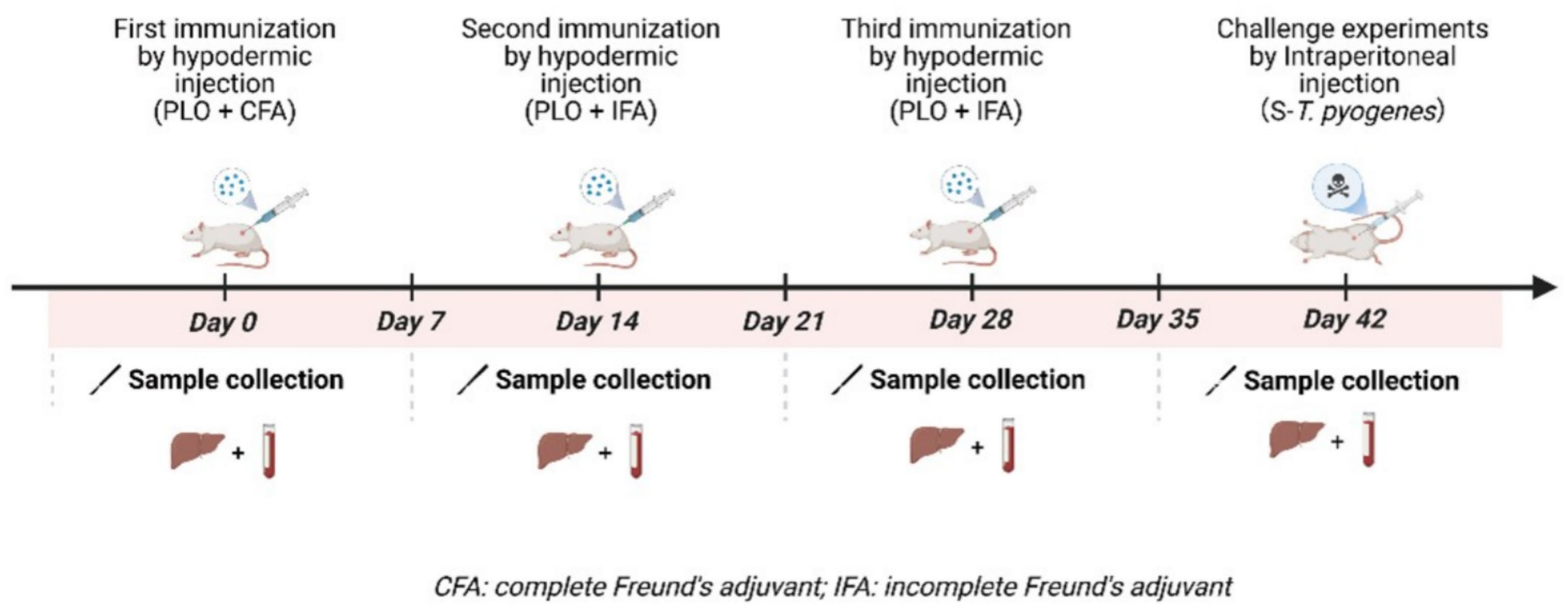
Schematic timeline of immunogen administration and challenge. This figure was created with BioRender (https://biorender.com/).

Mice with an average initial body weight of 20 ± 2 g were randomly allocated into 4 groups (22 mice per group) using a completely randomized design: (1) D-rPLO (deer-derived) group, (2) S-rPLO (swine-derived) group, (3) positive control group, and (4) negative control group. The experimental group received first immunization with two immunogens emulsified in Freund’s complete adjuvant, followed by second and third immunizations using the same immunogens in Freund’s incomplete adjuvant. The positive control group was injected with PBS following the same regimen, while the negative control group received no intervention. The rPLO antigen was diluted in PBS to an optimal concentration and emulsified with Freund’s adjuvant in a 1:1 volume ratio to prepare the immunogen. Subcutaneous immunizations were administered at multiple dorsal sites on days 0, 14, and 28, with each mouse receiving a 100 μg dose of rPLO. Blood and spleen samples were collected on days 0, 7, 21, and 35 for subsequent analyses (Figure 1).

### 2.8 Detection of *Trueperella pyogenes*-specific antibody levels in serum

Serum IgG levels against PLO-specific antigens were measured by indirect ELISA. Purified D-rPLO (8 μg/mL in carbonate coating buffer) was coated onto 96-well ELISA plates (100 μL/well) and incubated overnight at 4 °C. After three washes with PBST, wells were blocked with 4% BSA (200 μL/well) at 37 °C for 2 h. Serum samples diluted 1:400 in PBS were added (100 μL/well) and incubated at 37 °C for 1 h. Following another washing cycle, horseradish peroxidase (HRP)-conjugated goat anti-mouse IgG (1:6000, 100 μL/well) was added and incubated at 37 °C for 45 min. After final washing, TMB substrate (100 μL/well) was developed for 15 min at room temperature before adding stop solution (50 μL/well). Absorbance was measured at 450 nm using a microplate reader.

### 2.9 Analysis of CD4^+^/CD8^+^ markers and cytokine mRNA expression in splenocytes

Single-cell suspensions were prepared from spleens, and cell viability was determined by Trypan Blue exclusion. Cells (1 × 10^6^ cells/mL) were incubated with anti-mouse CD3-FITC, CD4-APC, and CD8-PE antibodies (5 μL per antibody) at 4 °C for 30 min. Following washing steps, CD4^+^ and CD8^+^ T cells were subjected to flow cytometric analysis using a BD FACSCanto II instrument.

Total RNA was isolated from splenocytes for quantitative PCR (qPCR) analysis. First-strand cDNA synthesis was performed using HiScript II Q RT SuperMix (Vazyme) under recommended conditions. Gene-specific primers for IL-2, TNF-*α*, IL-10, and *β*-actin (sequences listed in Table 1) were designed based on published references (29–32). Quantitative amplification was carried out on an Applied Biosystems QuantStudio 5 Real-Time PCR System with the following cycling parameters: 95 °C for 30 s, 40 cycles of 95 °C for 5 s, and 60 °C for 34 s. Relative mRNA expression levels were calculated using the 2^−ΔΔCT^ method, with *β*-actin as the endogenous control.

### 2.10 Challenge protection assay

Fourteen days after the third immunization, all experimental groups were intraperitoneally challenged with 5 × LD_50_ of S-*T. pyogenes* isolates in a 0.2 mL dose per mouse. Survival was monitored daily for 14 days post-challenge. Following euthanasia, lung and liver tissues were collected, fixed in 10% neutral buffered formalin, embedded in paraffin, sectioned (5 μm thickness), and stained with hematoxylin and eosin (H&E) for histopathological evaluation.

### 2.11 Statistical analysis

Statistical analyses were performed using GraphPad Prism 9.0 (San Diego, CA, USA), with all graphs generated through this platform. Data are expressed as the mean ± standard error of the mean (SEM). Flow cytometry data were analyzed with FlowJo software (v10.8; BD Biosciences, San Jose, CA, USA). Statistical significance was assessed by two-way ANOVA with the following notation: ns (not significant), *p < 0.05, **p < 0.01, and ***p < 0.001.

## 3 Results

### 3.1 Morphological and molecular identification and analysis of isolates

**Figure 2:**
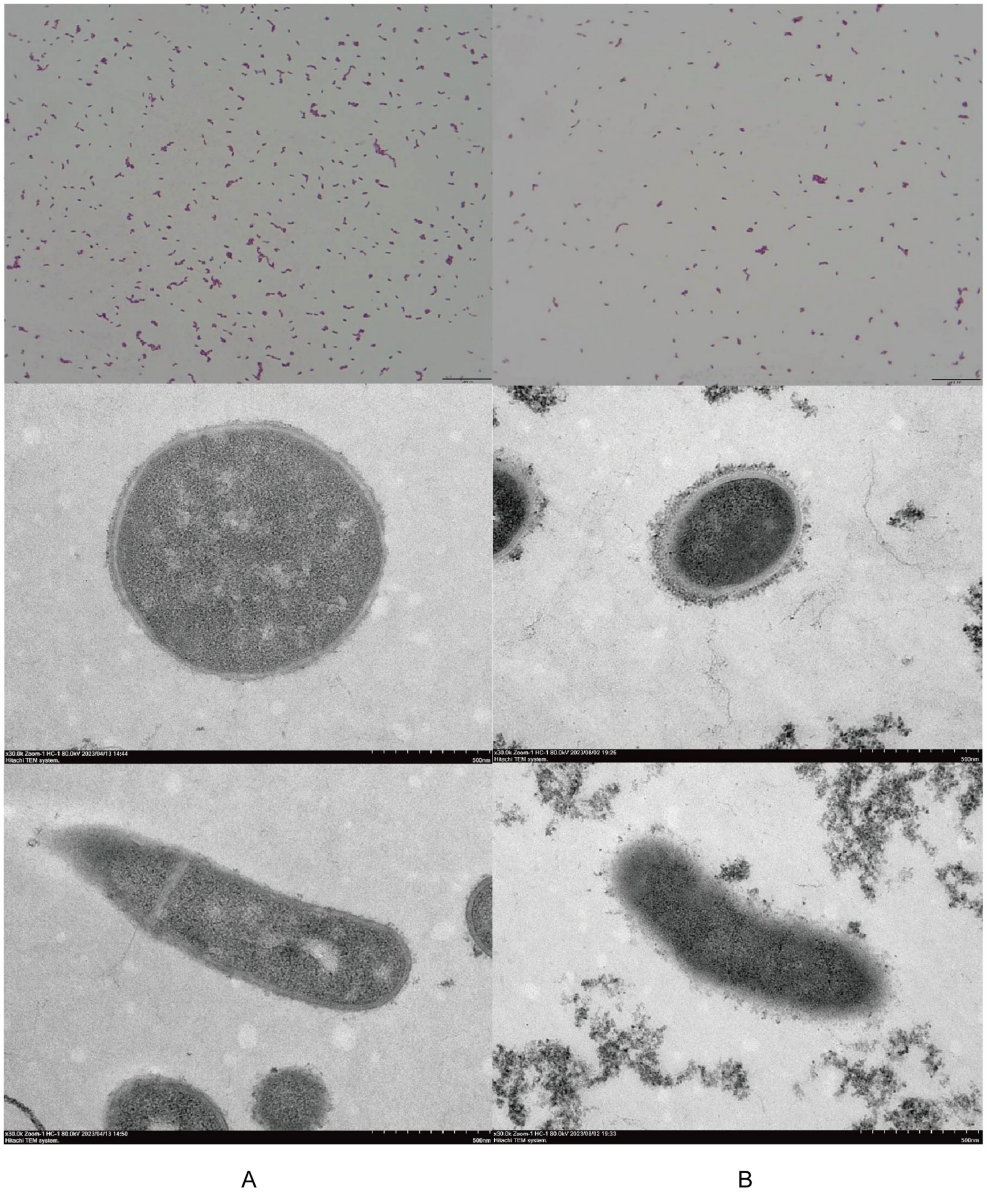
Microscopic examination of two isolated strains. Comparison of the morphology of isolates from deer **(A)** and swine **(B)** using Gram staining (1,000× magnification) and transmission electron microscopy (TEM, 30,000× magnification). From top to bottom: Gram stain images, TEM views perpendicular to the bacteria’s long axis, and TEM views parallel to the bacteria’s long axis.

Gram staining indicated that both purified isolates were Gram-positive short rods, although they displayed morphological differences between the strains. TEM further showed that neither isolate possessed spores, capsules, or flagella (Figures 2A,B).

Amplification and sequencing of the 16S rRNA gene produced a 1,465-bp fragment. BLAST comparison with GenBank sequences revealed over 98% similarity to *T. pyogenes*, confirming their identification as *T. pyogenes* despite the morphological variations observed. The phenotypic characteristics of the two *T. pyogenes* strains are presented in Supplementary Figures S1, S2. The isolates were named D-*T. pyogenes* (originating from deer) and S-*T. pyogenes* (originating from swine), respectively.

### 3.2 Pathogenicity analysis

The LD_50_ in mice was found to be 6.8 × 10^10^ colony-forming units (CFU) for D-*T. pyogenes* and 2.3 × 10^7^ CFU for S-*T. pyogenes* (Supplementary Tables S2, S3). This indicates a significant difference in virulence between the strains, with S-*T. pyogenes* showing about 3,000 times higher pathogenicity.

### 3.3 Genomic sequencing and structural analysis of PLO

The complete genome sequences of two *T. pyogenes* strains have been submitted to GenBank with accession numbers JARFUR000000000 (D-*T. pyogenes*) and JBBVUN000000000 (S-*T. pyogenes*), as summarized in Table 2. Analysis using the VFDB database identified 55 virulence genes across 13 functional categories in the deer-derived strain (D-*T. pyogenes*), whereas the swine-derived strain (S-*T. pyogenes*) exhibited a broader virulence gene profile, containing 57 genes spanning 15 categories. Notably, S-*T. pyogenes* possessed additional genes linked to nutritional toxicity and specialized secretion systems, as detailed in Table 3.

**Table 2:** Whole genome sequencing data for two *T. pyogenes* strains.

Item	D-*T. pyogenes*	S-*T. pyogenes*
Contigs	489	152
Largest contig (bp)	40,002	71,060
Total length (bp)	2,270,251	2,298,721
GC%	59.64	59.51
N50 (bp)	6,939	30,835
N75 (bp)	4,097	14,863
L50	100	26
L75	203	53

N50: sequences are arranged from longest to shortest, and the length of the sequence at which the cumulative length reaches 50% of the total is the N50 value.

N75: sequences are similarly ordered, and the length of the sequence at which the cumulative length reaches 75% of the total is the N75 value.

L50: the count of contigs needed to reach the N50 length.

L75: the count of contigs needed to reach the N75 length.

**Table 3:** Annotation results of virulence factors in *T. pyogenes* from deer and swine based on the VFDB database.

Virulence gene class	D-*T. pyogenes*		S-*T. pyogenes*	
	Type	Number	Type	Number
Adherence	5	18	5	18
Iron uptake	4	15	4	15
Regulation	6	6	6	6
Toxin	2	2	2	2
Amino acid and purine metabolism	1	1	1	1
Anti-apoptosis factor	1	1	1	1
Antiphagocytosis	1	1	1	1
Cell surface components	2	2	2	2
Immune evasion	2	3	2	3
Lipid and fatty acid metabolism	1	2	1	2
Nutritional virulence	0	0	1	1
Phagosome arresting	1	1	1	1
Protease	2	2	2	2
Secretion system	0	0	1	1
Stress adaptation	1	1	1	1

**Figure 3:**
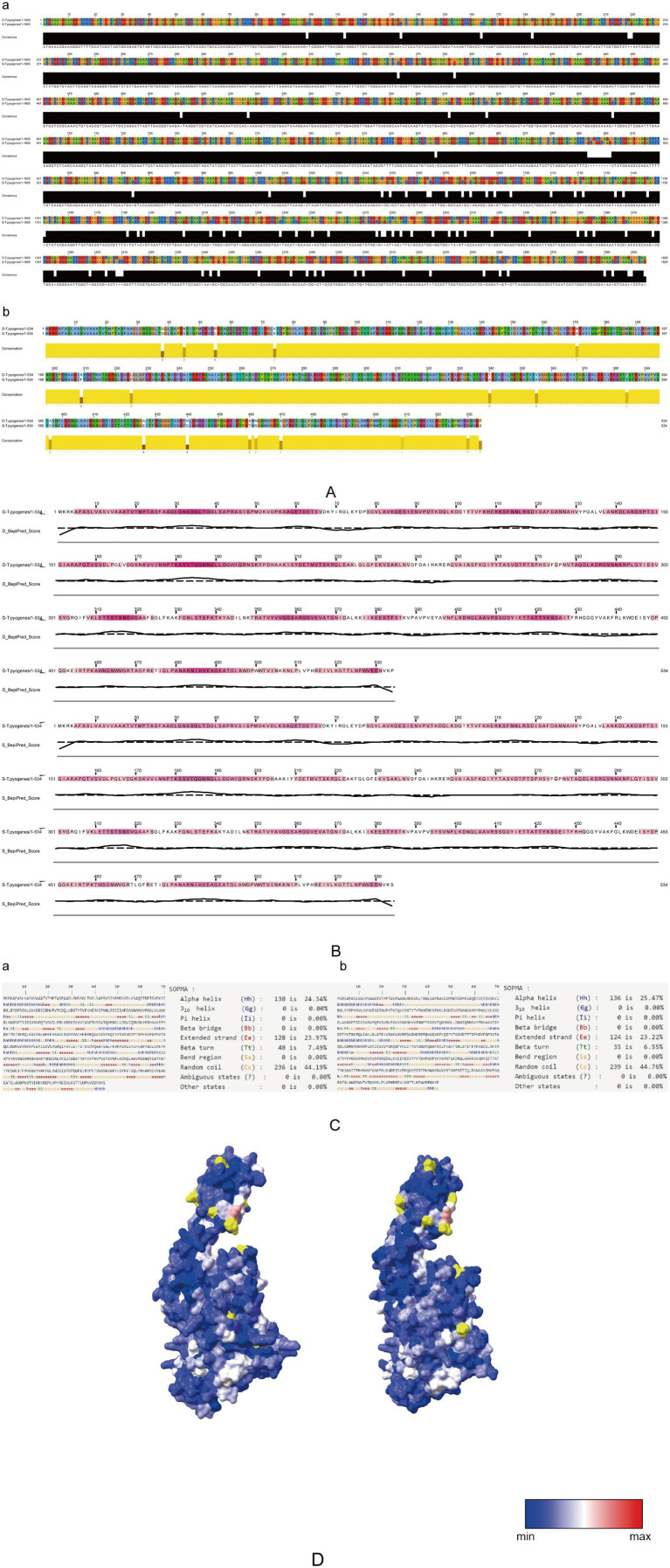
Bioinformatics comparative analysis of the virulence factor PLO. **(A)** Alignment of nucleotide sequences (a) and amino acid sequences (b) of *T. pyogenes* isolates from swine and deer, performed with ClustalX 2.0 and visualized using Jalview. **(B)** Antigenic epitopes of swine- and deer-derived *T. pyogenes* were predicted using BepiPred-2.0 and visualized with Jalview, with a threshold of 0.5. **(C)** Comparison of the predicted secondary structures of PLO from *T. pyogenes* strains derived from swine (a) and deer (b), analyzed with SOPMA. **(D)** Comparison of the predicted tertiary structures of PLO from *T. pyogenes* strains derived from deer (left) and swine (right), generated using Phyre 2, BepiPred-2.0, and ChimeraX.

A comparative examination of the virulence-associated *plo* gene from *T. pyogenes* isolates originating from deer and swine revealed differences in both structure and function. Multiple sequence alignment analysis was conducted using Jalview. The *plo* gene sequences differed by 85 nucleotides, resulting in 94.7% sequence identity, which corresponded to 19 amino acid substitutions and 96.4% identity at the protein level (Figure 3A). Antigenic epitope composition predicted by BepiPred-2.0 showed discernible differences between the two variants (Figure 3B). Secondary structure analysis indicated distinct patterns: the deer-derived PLO (D-PLO) comprised 25.5% *α*-helices (136 residues), 6.6% *β*-sheets (35 residues), 44.8% random coils (239 residues), and 23.2% extended strands (124 residues). In contrast, the swine-derived PLO (S-PLO) showed a slightly lower α-helical content (24.3%, 130 residues) but higher proportions of β-sheets (7.5%, 40 residues) and extended strands (24.0%, 128 residues) (Figure 3C). Three-dimensional structural modeling using the highest-scoring templates yielded greater confidence for D-PLO, covering 483 residues (90% sequence coverage at 100% confidence), compared to 461 residues (86% coverage) for S-PLO (Figure 3C). The specific distribution of variant residues is shown in Table 4. The results indicate that the number of non-synonymous mutations between the two strains is limited, and the majority of these mutations are located in buried, interior regions of the protein. This suggests that PLO may have evolved primarily under constraints to maintain structural stability, rather than through extensive surface remodeling to achieve functional divergence (Figure 3D).

**Table 4:** Distribution of variant sites within the protein.

No.	Mutation site	D-*T. pyogenes*		S-*T. pyogenes*	
		Predicted antigenic site (E)	Spatial location (B: buried/E: exposed)	Predicted antigenic site (E)	Spatial location (B: buried/E: exposed)
1	G38D	E	B	E	B
2	A45V	E	B	E	B
3	P55L		B		B
4	K74E		B		B
5	N171S	E	E	E	E
6	S209F	E	E		E
7	L225F		B		B
8	T340A	E	B	E	B
9	V355A	E	B	E	B
10	F384Y	E	B	E	B
11	A396S		B	E	B
12	A426E	E	B	E	B
13	R440G		B		B
14	A460T	E	B	E	B
15	N462S	E	E	E	E
16	A470L	E	B		B
17	L509I	E	B	E	B
18	E530D	E	B	E	B

### 3.4 Expression of the recombinant PLO proteins

**Figure 4:**
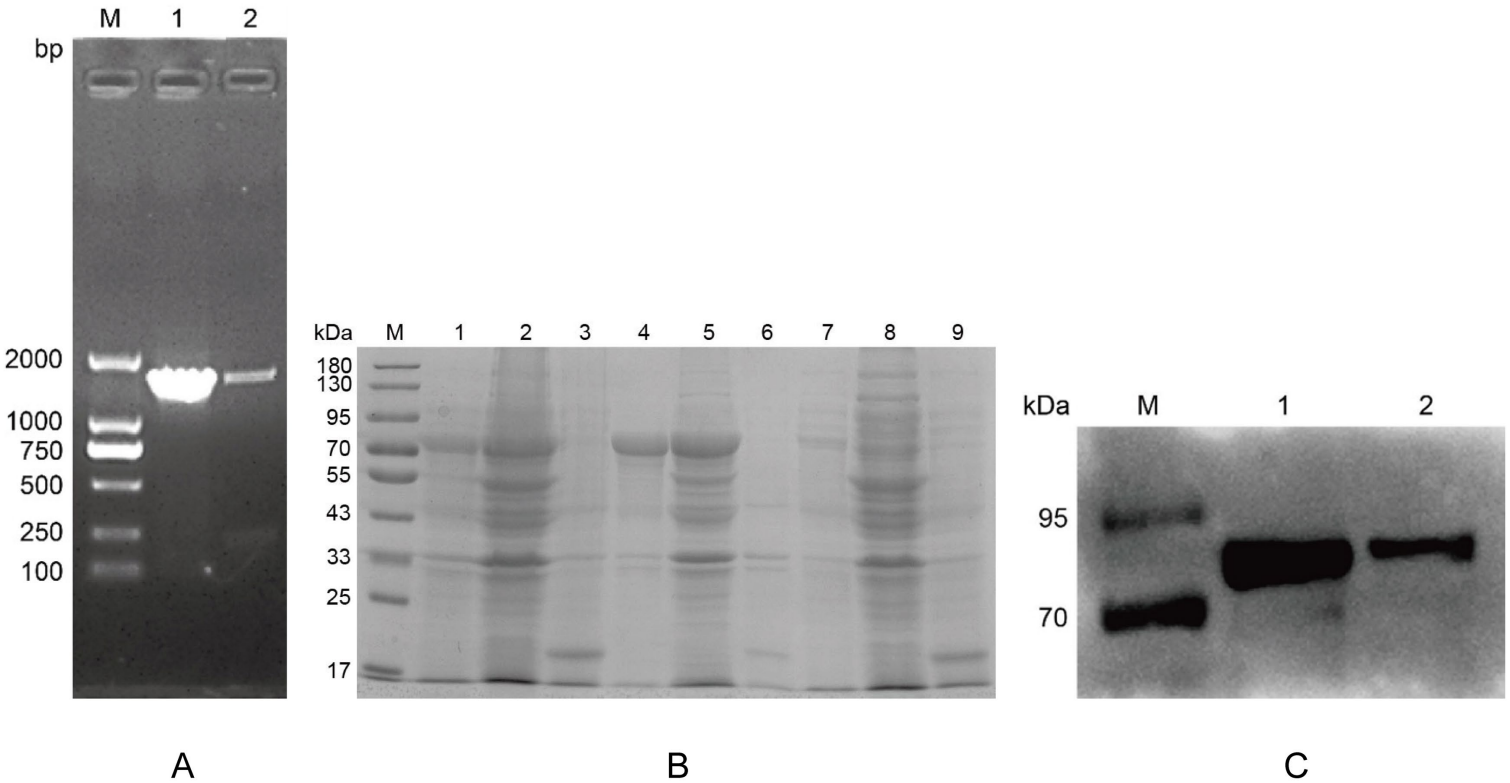
Expression and identification of recombinant PLO protein in *E. coli* BL21. **(A)** PCR amplification results of *T. pyogenes* PLO genes. Lane M: DL2000 marker; Lane 1: product of deer-derived *T. pyogenes* PLO gene; Lane 2: product of swine-derived *T. pyogenes* PLO gene. **(B)** SDS-PAGE analysis of recombinant PLO proteins. Lane 1: induced whole-cell lysate of deer-derived recombinant PLO (D-rPLO); Lane 2: induced whole-cell lysate of swine-derived recombinant PLO (S-rPLO); Lane 3: induced whole-cell lysate containing pET32a vector only; Lane 4: pellet fraction from D-rPLO lysate; Lane 5: pellet fraction from S-rPLO lysate; Lane 6: pellet fraction from pET32a lysate; Lane 7: supernatant from D-rPLO lysate; Lane 8: supernatant from S-rPLO lysate; Lane 9: supernatant from pET32a lysate. **(C)** Western blot detection of recombinant PLO proteins. Lane 1: D-rPLO protein; Lane 2: S-rPLO protein.

The *plo* genes from D-*T. pyogenes* and S-*T. pyogenes* were cloned (Figure 4A) and successfully expressed in *E. coli*. SDS-PAGE analysis demonstrated the expression of His-tagged rPLO with molecular weights of 74.44 kDa (D-rPLO) and 77.27 kDa (S-rPLO), which predominantly localized in inclusion bodies (Figure 4B). Western blot analysis using an anti-His antibody confirmed the specificity of the recombinant proteins (Figure 4C).

### 3.5 Serum PLO-specific antibody levels

**Figure 5:**
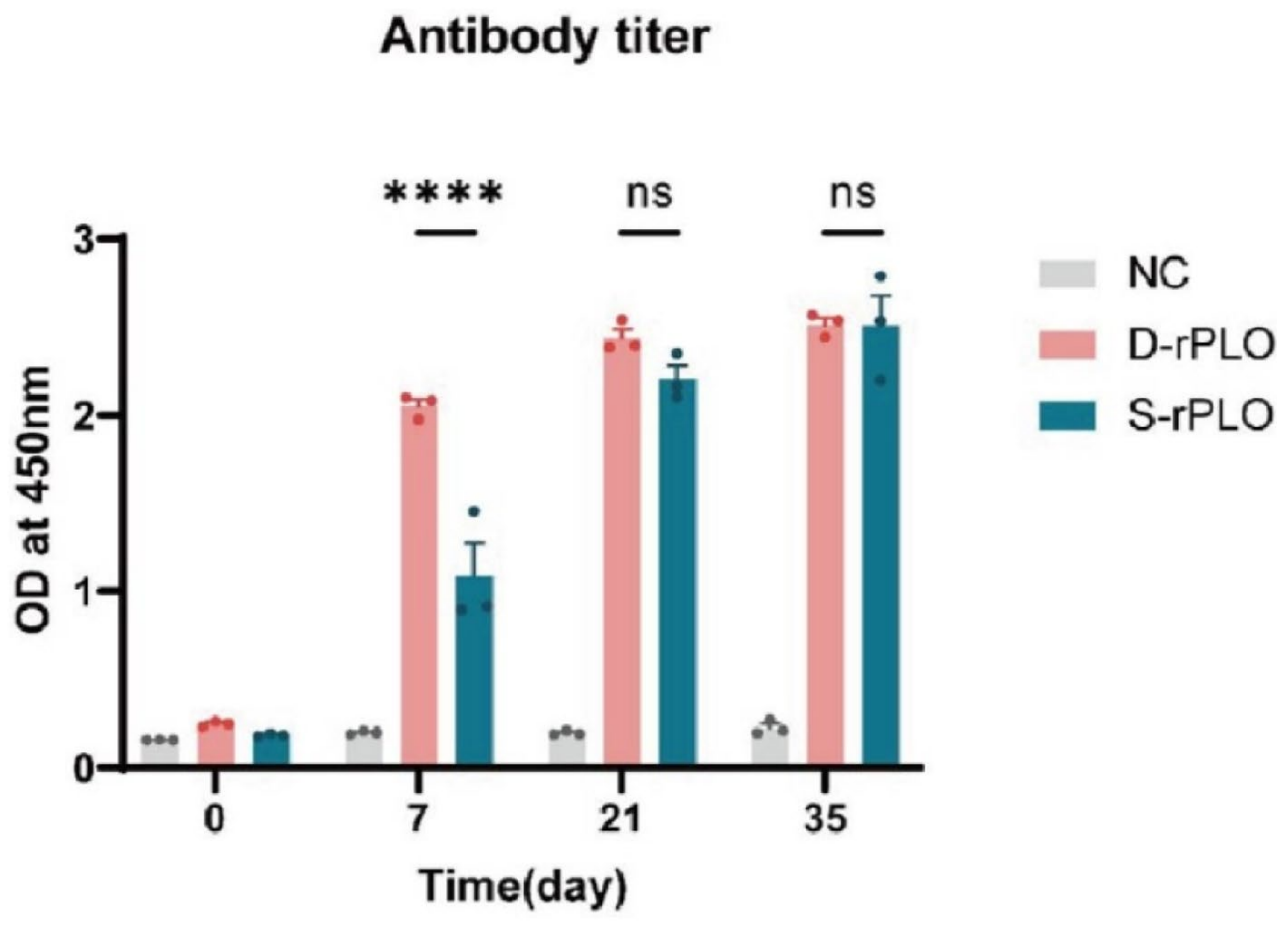
Serum PLO-specific antibody levels. The levels of the PLO-specific antibody were detected by indirect ELISA.

Immunization using rPLO combined with Freund’s adjuvant triggered strong antibody responses. Both the D-rPLO and S-rPLO vaccinated groups achieved their highest IgG levels by day 35, which were significantly greater than those in the negative control group (*p* < 0.01). While D-rPLO produced higher antibody titers on day 7 (*p* < 0.01), antibody levels in both groups were similar on days 21 and 35 (Figure 5).

### 3.6 Comparative analysis of CD4^+^, CD8^+^ T cells and the CD4^+^/CD8^+^ ratio triggered by PLO proteins

**Figure 6:**
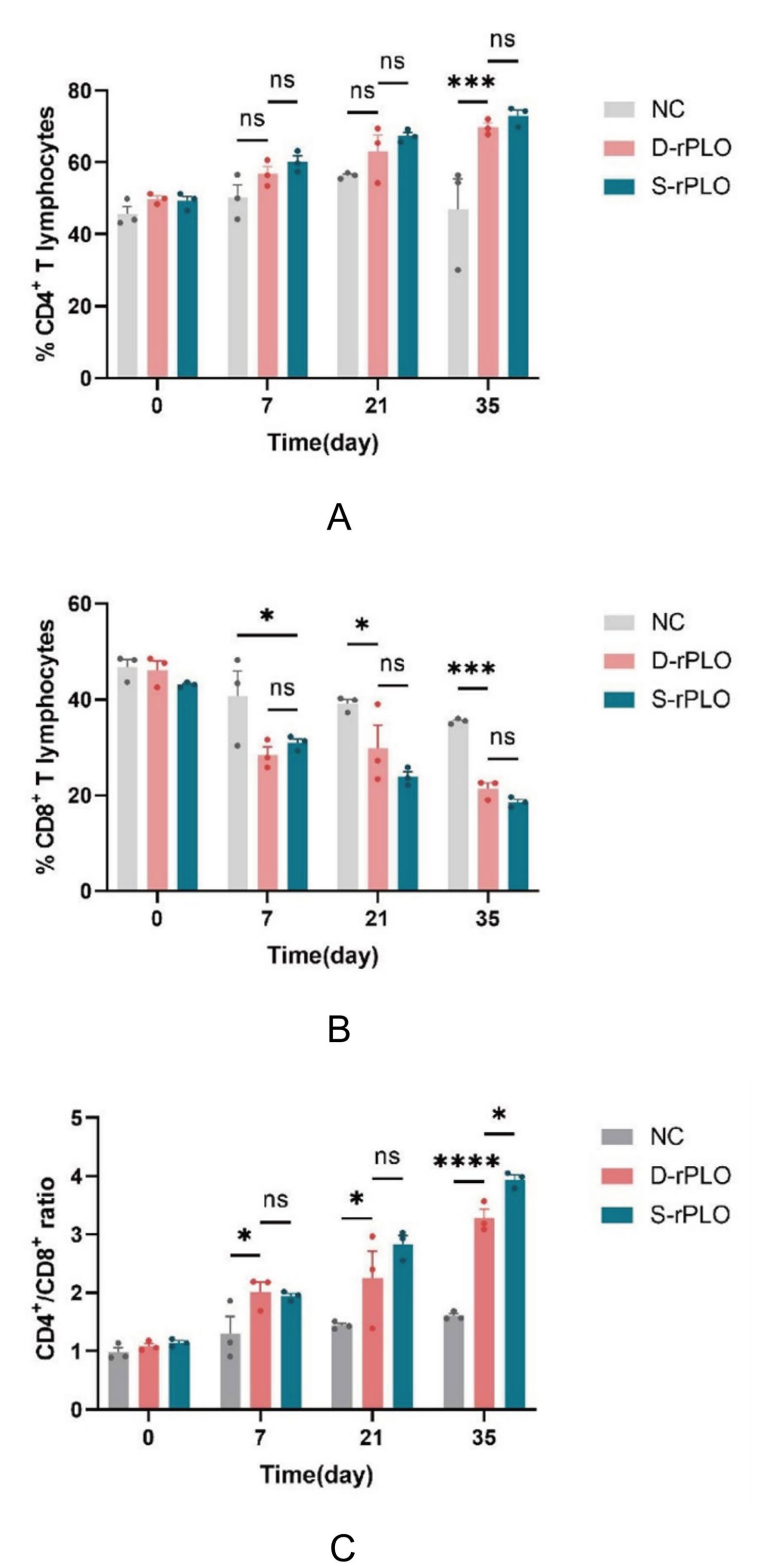
Cellular immune responses triggered by the PLO proteins. Results of CD4^+^ **(A)** and CD8^+^ **(B)** T-lymphocyte expression in the spleen of mice from different immunization groups. **(C)** The ratio of CD4^+^/CD8^+^ T-lymphocyte expression in spleen of mice.

Flow cytometry analysis of splenic lymphocyte subsets revealed a dominant proliferation and functional predominance of CD4^+^ T cells. The proportion of CD4^+^ T cells steadily increased across all immunized groups. A highly significant difference (*p* < 0.001) was noted between the immunized groups and the negative control group on day 35. While no statistically significant differences were found among the immunized groups themselves (Figure 6A). Conversely, the percentage of CD8^+^ T cells gradually decreased. Significant differences (*p* < 0.05) were observed between the immunized groups and the negative control group on days 7 and 21, becoming highly significant (*p* < 0.001) by day 35. No significant differences were detected among the immunized groups (Figure 6B). The CD4^+^/CD8^+^ ratio increased progressively after immunization, with significant differences between the immunized groups and the negative control on days 7 (*p* < 0.05) and 21 (*p* < 0.05), reaching a highly significant difference by day 35 (*p* < 0.01). Although the D-rPLO and S-rPLO groups had similar ratios on days 7 and 21, the S-rPLO group exhibited a higher CD4^+^/CD8^+^ ratio than the D-rPLO group at day 35 (*p* < 0.05) (Figure 6C).

### 3.7 Comparative analysis of cytokine expression dynamics induced by PLO proteins using qRT-PCR

**Figure 7:**
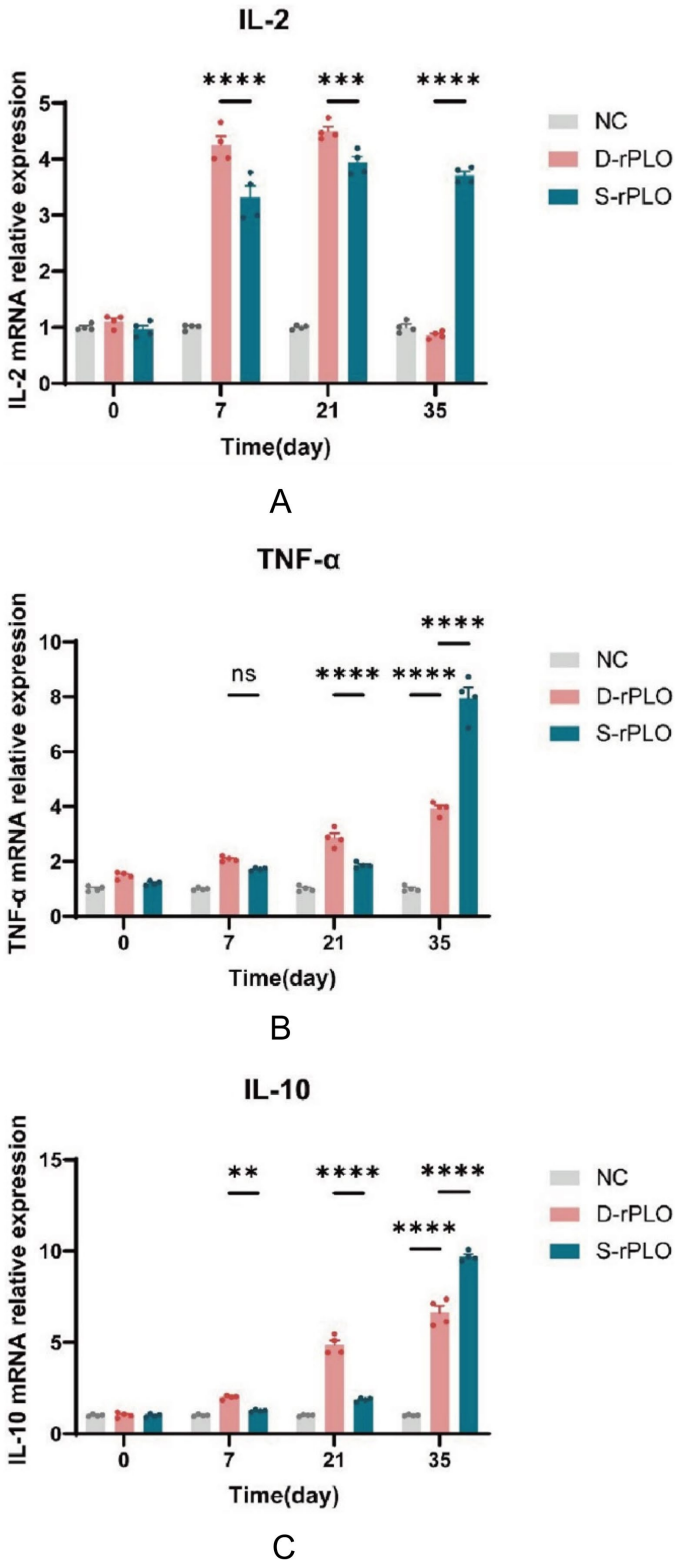
Transcription levels of mice splenic lymphocyte factors. Quantification of IL-2 **(A)**, TNF-*α* **(B)**, and IL-10 **(C)** expression in splenic cells using qRT-PCR.

To characterize the changes in cytokine expression, qRT-PCR was employed to measure levels of both pro-inflammatory cytokines (IL-2, TNF-*α*) and the anti-inflammatory cytokine (IL-10). During the early period (days 7 to 21), the D-rPLO formulation showed significantly higher expression of IL-2, TNF-α, and IL-10 compared to S-rPLO, with the most notable differences observed on day 21 (IL-2, *p* < 0.001; TNF-α, *p* < 0.0001; IL-10, *p* < 0.0001) (Figures 7A,B). However, by day 35, this trend completely reversed, with S-rPLO displaying significantly greater expression of all three cytokines than D-rPLO, with highly significant statistical values (*p* < 0.0001) (Figure 7C).

### 3.8 Analysis of protective effectiveness of recombinant PLO proteins in immunized mice

**Figure 8:**
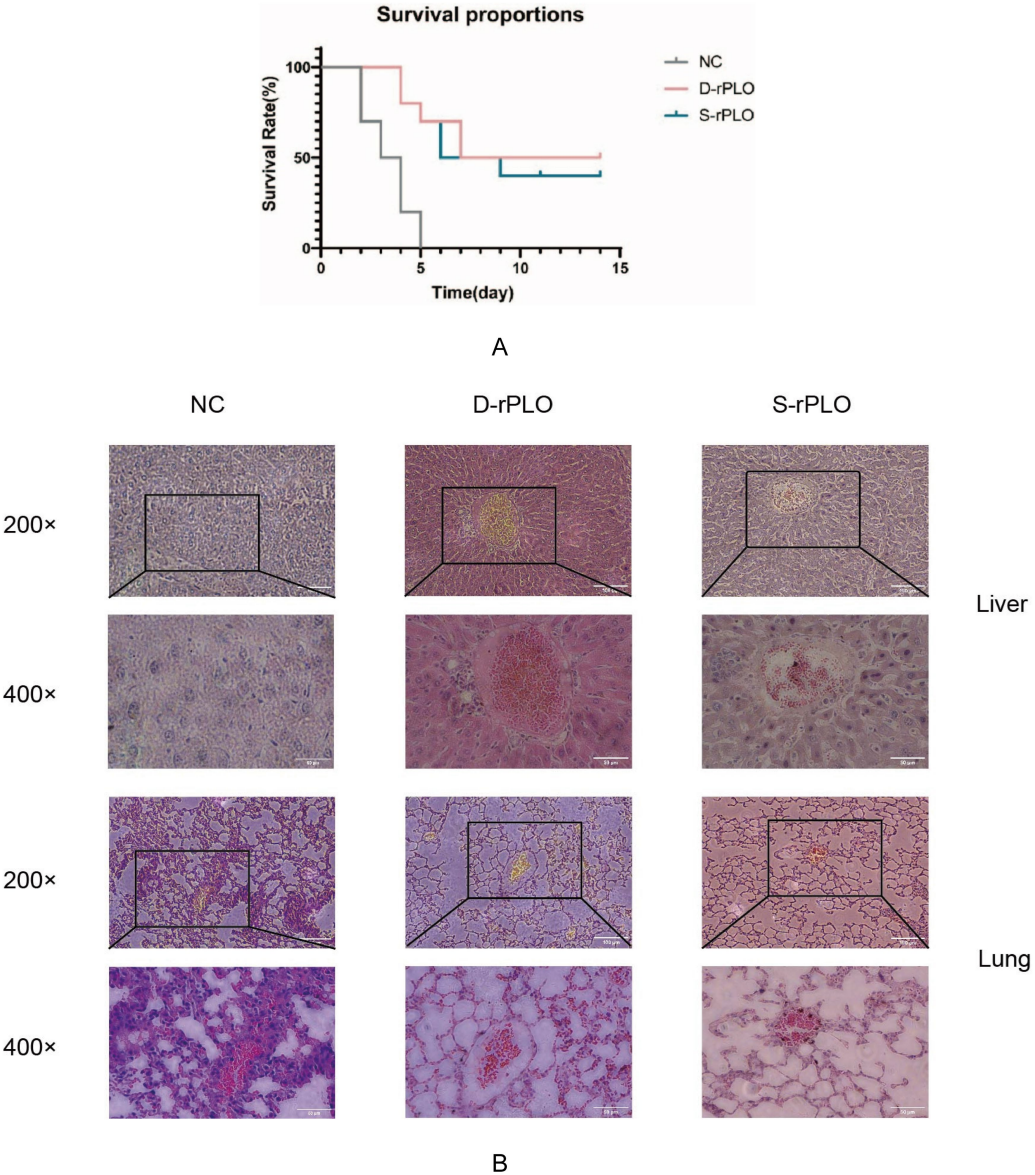
Survival analysis and tissue pathology in challenged mice. **(A)** Survival curves of mice post-challenge. **(B)** Histopathological examination of H&E-stained liver and lung tissues. Tissues were collected post-challenge, stained with H&E, and examined under a light microscope to assess and compare the histopathological changes.

To assess the protective effectiveness of recombinant proteins from different sources, mice immunized with either D-rPLO or S-rPLO antigens were exposed to a virulent S-*T. pyogenes* strain at 5 × LD_50_. Survival analysis showed partial protection in both vaccinated groups, with 50% survival in the D-rPLO group and 40% in the S-rPLO group, compared to 100% mortality in the unvaccinated control group (Figure 8A).

Postmortem histopathological examination of liver and lung tissues revealed significant differences between groups (Figure 8B). In the control group, liver sections showed disrupted architecture with loss of hepatocyte monolayers, widespread coagulative necrosis, and marked inflammatory cell infiltration in the portal triads. Lung pathology included alveolar epithelial hyperplasia (proliferation of type II pneumocytes), thickening of interstitial septa, and distortion of airspaces. In the S-rPLO group, liver lobules preserved their radial plate arrangement around central veins and maintained sinusoidal structure, with only localized inflammatory clusters. Lung tissue showed mild bronchiolar epithelial hyperplasia and minimal perivascular lymphocyte infiltration. In the D-rPLO group, hepatocytes exhibited normal polygonal shape with intact cytoplasmic and nuclear features. Lung sections displayed uniformly thin alveolar septa, although some localized vascular congestion and capillary dilation were observed.

## 4 Discussion

Using a combination of morphological examination and 16S rRNA gene sequencing, the two strains isolated in this study were identified as *T. pyogenes*. Building on previous research that highlighted the effectiveness of bioinformatics in uncovering pathogenic mechanisms and evolutionary patterns through genomic analysis of virulence factors, we conducted a comparative genomic study of the two *T. pyogenes* isolates. By combining whole-genome sequencing with advanced bioinformatics techniques, we focused on analyzing their genetic makeup and structural differences in the *plo* gene. The comparison revealed notable variations in the virulence gene profiles between the strains. Specifically, the S-*T. pyogenes* strain contained unique virulence factors, such as nutritional toxicity and specialized secretion systems, which were absent in the D-*T. pyogenes* strain. Further comparison of the *plo* gene sequences showed 85 nucleotide differences (94.7% sequence identity) and 19 amino acid variations (96.4% identity) in the corresponding proteins. Among the 19 variant residues, S209F, A396S, and A470L exhibited distinct epitope characteristics between the two strains. Although the S209F substitution is located adjacent to a predicted antigenic region in the D-*T. pyogenes* structure, it does not represent a stably exposed site in the S-*T. pyogenes* protein. The substitution from serine to phenylalanine markedly increases side-chain volume and hydrophobicity, which may promote local hydrophobic packing and lead to partial residue retraction or masking within the three-dimensional conformation. These results suggest that S209F in S-*T. pyogenes* is more likely involved in conformational or structural regulation rather than direct antigen recognition. In contrast, the A396S substitution displayed clear antigenic relevance in S-*T. pyogenes*. This residue is located in a stably exposed region on the protein surface and is consistently predicted as a B-cell epitope in the S-*T. pyogenes* conformation. The alanine-to-serine replacement introduces a polar hydroxyl group, potentially altering the local hydrogen-bonding network and reshaping the microarchitecture of the epitope. The A470L substitution is positioned within the hydrophobic core of the protein and does not constitute an antigenic site in S-*T. pyogenes*. Replacement of alanine with leucine increases hydrophobic side-chain volume, thereby enhancing internal packing density and overall structural rigidity. Such internal stabilizing mutations are commonly associated with improved protein folding efficiency, secretion stability, or prolonged functional persistence *in vivo*, and may structurally support the enhanced virulence of S-*T. pyogenes*. Taken together, these findings indicate that the virulence of PLO may be associated with internal structural stability as well as selective alterations of a limited number of surface residues. The coordinated interplay between internal stabilization and surface immunogenic modulation may represent a key molecular mechanism underlying the high-virulence phenotype. These genetic differences partially explain the increased virulence of S-*T. pyogenes* in mice. However, challenge experiments showed that S-*T. pyogenes* was nearly 1,000 times more pathogenic than D-*T. pyogenes*—a gap far exceeding what virulence factor abundance alone can explain. We therefore focused on PLO, the major virulence determinant in *T. pyogenes*. Moreover, the genetic diversity in the *plo* gene linked to different host origins suggests distinct evolutionary adaptations, resulting in varied immunogenicity and protective effects of the bacterial isolates (24, 33, 34). This observation is consistent with previous studies reporting host-specific genomic flexibility in pathogenic bacteria (27, 35, 36).

Humoral and cellular immunity are essential markers for assessing vaccine immunogenicity. In this study, mice vaccinated with rPLO antigens showed a marked increase in serum-specific antibodies. Importantly, the D-rPLO group had higher antibody levels than the S-rPLO group after the initial immunization, although this difference lessened after booster shots. These results indicate that both recombinant proteins successfully triggered strong antibody responses, with D-rPLO eliciting a faster immune activation during the early stages of immunization.

CD4^+^ and CD8^+^ are transmembrane glycoproteins mainly found on T lymphocytes. Acting as coreceptors in cell-mediated immunity, CD4^+^ specifically recognizes antigen peptides presented by MHC class II molecules, whereas CD8^+^ binds to peptide complexes presented by MHC class I (37). The ratio of CD4^+^ to CD8^+^ cells is an important biomarker used to assess immune status and functional balance in clinical immunology. Recent research into the pathogenesis of *T. pyogenes* infections has emphasized the vital role of host immune responses in clearing the bacteria. The pathogen is primarily removed by phagocytic cells, which gradually decrease its viability over time. Importantly, immunity mediated by CD4^+^ T cells, especially the Th1 subtype, has been identified as a key mechanism in controlling *T. pyogenes* infection (20, 21). Th1 cells promote strong inflammatory responses and enhance the activation of M1 macrophages, which are essential for eliminating intracellular pathogens (38, 39). These observations are consistent with flow cytometry results from the current study, which showed a significant increase in the CD4^+^/CD8^+^ ratio after immunization, suggesting predominant activation of CD4^+^ T cell-dependent pathways. However, a notable difference from earlier work by Huang (21) was found regarding CD8^+^ T cell responses. While their study reported significant proliferation of CD8^+^ T cells following immunization, our findings did not show a considerable increase in this subset. This discrepancy may be due to variations in vaccine design and antigen presentation pathways. In this study, a chimeric gene DNA vaccine encapsulated in chitosan nanoparticles was used. DNA vaccines typically promote endogenous antigen processing through MHC class I molecules, theoretically priming CD8^+^ T cells. Conversely, the recombinant PLO protein component likely follows the exogenous pathway via MHC class II, mainly activating CD4^+^ T cells. This dual mechanism could explain the dominant CD4^+^ response and the limited expansion of CD8^+^ T cells observed here.

To gain deeper insight into the immune response dynamics, we measured the relative expression levels of key cytokines (IL-2, TNF-*α*, and IL-10) using qRT-PCR after immunization with recombinant PLO proteins. In line with earlier research (21, 22), both D-rPLO and S-rPLO significantly increased the expression of pro-inflammatory cytokines (IL-2, TNF-α) as well as the anti-inflammatory cytokine IL-10, indicating a coordinated immune defense against *T. pyogenes*. Importantly, D-rPLO triggered a more rapid rise in IL-2 and TNF-α levels compared to S-rPLO. IL-2, mainly produced by antigen-activated CD4^+^ T cells, plays a crucial role in initiating Th1-type immune responses and boosting macrophage-mediated cytotoxicity (40, 41). Likewise, low levels of TNF-α support Th1 differentiation and enhance cellular immunity (42). These results support the idea that strong Th1 activation is essential for fighting intracellular pathogens such as *T. pyogenes*.

Notably, although the S-rPLO group showed increased IL-2 and TNF-α levels after the third booster immunization, the D-rPLO group showed superior survival rates post-challenge. This discrepancy may be explained by the strong pore-forming activity of the more virulent S-rPLO during the initial immunization. This potent cytotoxicity may cause excessive membrane disruption and necrosis in immune cells, which could explain the absence of an early, rapid surge in cytokine production following initial immunization with S-rPLO (43). With repeated immunizations, however, the host progressively develops an adaptive immune background against PLO. The production of neutralizing antibodies and memory immune cells attenuates the acute cytotoxicity of PLO, thereby reducing its inflammatory stimulus to an optimal range. As a result, by the third immunization, the S-rPLO efficiently induces a substantial rise in cytokine production. In contrast, the less virulent deer-origin PLO triggers a more moderate cytokine response. Its limited amplification effect in later stages may be due to its lower inherent virulence and reduced stimulatory capacity. Therefore, the key to designing an effective vaccine lies in eliciting a robust immune response while minimizing cytotoxicity caused by the excessive virulence of the immunogen. Previous research (21) demonstrated that a chimeric DNA vaccine, constructed by combining multiple virulence-associated epitopes—such as *plo*, *cbpA*, *fimA*, and *nanH*—with CpG ODN 1826, successfully induced robust humoral and cellular immune responses in mice and provided significant protection against *T. pyogenes* infection. Thus, although virulence factors like CbpA, FimA, or NanH may not act as decisive protective antigens on their own, they may help optimize the immune environment, thereby promoting a rapid and high-level immune response following immunization with PLO.

The survival and histopathological findings together emphasize the varying protective effectiveness of the D-rPLO and S-rPLO vaccines against *T. pyogenes* infection. Although both recombinant antigens provided partial protection compared to unvaccinated controls, mice vaccinated with D-rPLO showed higher survival rates, which corresponded with histopathological signs of less tissue damage. This difference in protection is linked to significant genetic differences found in the *plo* gene sequences—85 nucleotide-specific variations (94.7% similarity) and 19 amino acid-specific differences (96.4% similarity) between host-adapted strains. These sequence variations suggest that *T. pyogenes* has undergone host-driven evolutionary changes, potentially modifying key epitopes that affect antigen recognition. Overall, these results suggest that the enhanced immunogenicity of D-rPLO may be due to its closer genetic match to the epitopes of the challenge strain, underscoring the importance of selecting the appropriate antigen source in vaccine development. Although the 3.6% difference in amino acid sequence appears small, it likely affects conformational epitopes critical for inducing protective immunity, which explains the differing clinical outcomes observed between the vaccinated groups. Based on bioinformatic analyses and previous studies on truncated PLO antigens and chimeric antigen vaccines, Yang et al. (19) functionally dissected the PLO protein and constructed two recombinant antigens: tPLOA1, containing amino acids 1–110 of PLO together with domain 4, and tPLOA2, containing amino acids 190–296 together with domain 4. These antigens were formulated with either Freund’s incomplete adjuvant or sheep red blood cell membranes and used to immunize mice. The results demonstrated that both formulations could enhance resistance to *T. pyogenes* infection to a certain extent, indicating that different regions of PLO harbor functional epitopes capable of inducing protective immune responses. However, a single truncated antigen was only able to confer limited protection. Furthermore, Hu et al. (18) fused domain 4 (D4) of *T. pyogenes* PLO with the C-terminal region of phospholipase C (PLC) from *Clostridium perfringens* to construct a chimeric protein, rPC-PD4. Immunization experiments showed that this chimeric antigen provided partial protection in mice, suggesting that the integration of immunodominant domains from different virulence factors or antigenic sources can enhance immune efficacy to some extent, although complete protection was not achieved. The PLO molecule contains multiple epitopes with immunogenic potential, which are distributed across different structural domains and spatial locations. In addition, surface-exposed residues differ from internal residues associated with protein stability, resulting in variations in antigen conformation, immune recognition, and virulence characteristics. Therefore, beyond traditional truncated or chimeric antigens, incorporating bioinformatically predicted units into vaccine design represents a promising strategy for developing multi-antigen or multi-epitope vaccines with broader coverage and higher protective potential. Such an approach may facilitate stable epitope exposure while simultaneously improving overall protein structural stability.

In summary, all these findings highlight the importance of developing multi-antigen vaccines and optimizing epitopes. The functional diversity of PLO between species enhances our understanding of host-specific bacterial pathogenesis and informs the design of targeted vaccines.

Inevitably, this study has several limitations. Although mice are a classical model in vaccine immunology and share a high degree of similarity in immune mechanisms with other animals, such as the functional conservation of Th1-type immune responses across species. However, the immune outcomes obtained in murine models should primarily be regarded as references for further investigation (44). Comparative immunology studies have shown that, for the parameters analyzed, the porcine immune system shares more than 80% similarity with that of humans, whereas the corresponding similarity in mice is only about 10%, highlighting the existence of pronounced species-specific differences (45). Similarly, among the principal natural hosts of *T. pyogenes*, ruminants exhibit a markedly higher proportion of *γδ* T cells in peripheral blood lymphocytes compared with mice, reaching up to 60%–70% (46, 47). This difference may substantially influence the development of protective immune responses. In addition, TLR9 expression and distribution in murine immune cell populations differ from those in other animal species, which has important implications for the use of synthetic oligodeoxynucleotides (CpG ODNs) as vaccine adjuvants (48). Therefore, although murine models provide a robust and mechanistically informative framework, validation in natural host species remains indispensable for a comprehensive assessment of translational relevance.

Moreover, in the research, the heterologous D-rPLO conferred better protection against challenge with S-*T. pyogenes* than the homologous S-rPLO. This phenomenon may be associated with strain-specific immunity and cross-protective immune responses. However, this study did not evaluate differences in cross-protective immunity under challenge with *T. pyogenes* strains originating from different hosts. Given the substantial strain diversity of *T. pyogenes* in natural populations, this issue is of considerable importance for understanding strain-specific versus cross-protective immunity and warrants systematic investigation in future studies.

## Data Availability

The datasets presented in this study can be found in online repositories. The names of the repository/repositories and accession number(s) can be found in the article/Supplementary material.
