# Supplementary material for: PLO genomic diversity underpins differential immunogenicity of Trueperella pyogenes strains from deer and swine
Source: Front Vet Sci. 2026 Mar 11;13:1758657. doi: 10.3389/fvets.2026.1758657 (PMC13013022; doi:10.3389/fvets.2026.1758657)
Supplement: Supplementary file 1 [file Data_Sheet_1.docx]

Supplementary Material

**PLO genomic diversity underpins differential immunogenicity of *Trueperella pyogenes* strains from deer and swine**


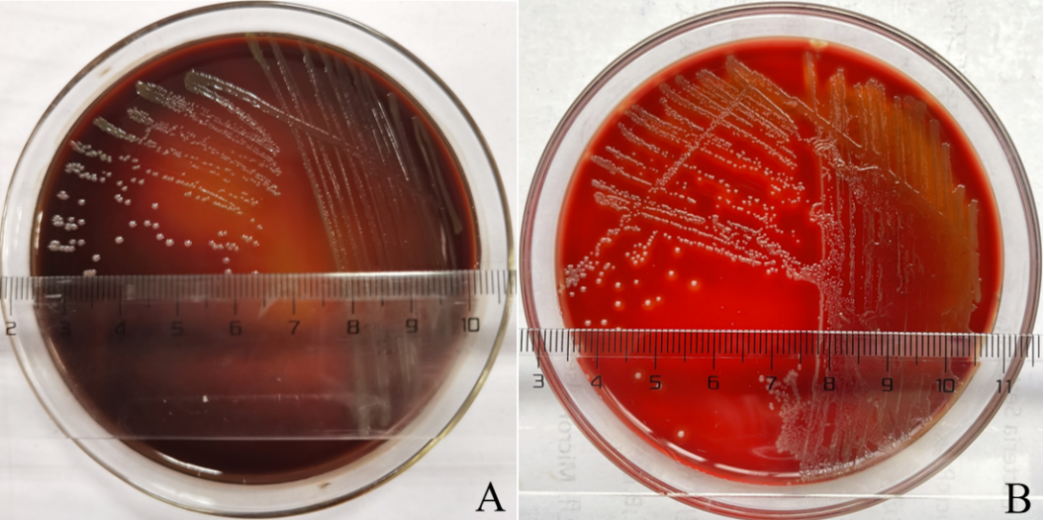


Supplementary Figure 1 A. Culture results of deer-derived isolates on rabbit blood plate; B. Culture results of swine-derived isolates on rabbit blood plate.

Samples collected from the suppurative lesions of affected sika deer and swine were inoculated onto 5% rabbit blood agar plates and incubated anaerobically at 37°C for 48 hours. Small, circular, white, translucent colonies were observed on the plates; these colonies were smooth, moist, convex, and possessed regular margins. Notably, a distinct zone of clear hemolysis was observed around the colonies, indicating a characteristic *β*-hemolytic phenotype (Supplementary Fig. 1).

Supplementary Table 1 Virulence gene primer sequences

| Primer Name | | sequence（5′-3′） | Product Length (bp) | Annealing Temperature (°C) |
| --- | --- | --- | --- | --- |
| PLO-g1-1 | F | AAAATGAGTCGTGCCAACATAG | 1286 | 57 |
|  | R | TCGTTTCCAGCTTGACAAAA |  |  |
| PLO-g1-2 | F | TAGTCAGTGGCGAAGGTAA | 1005 | 52 |
|  | R | CTGTTTGAAGGAAGCGATA |  |  |
| PLO-g2 | F | GGCCTTCTCGACGGTTGGAT | 1419 | 57 |
|  | R | TCGGCAAGTTCCTCGTGTCC |  |  |
| NanH | F | CGCTAGTGCTGTAGCGTTGTTAAGT | 781 | 57 |
|  | R | CCGAGGAGTTTTGACTGACTTTGT |  |  |
| NanP | F | AGCCATTCCGCCACTTTGGT | 791 | 55 |
|  | R | GATGAGTTGCTGGGAGGTCTTGC |  |  |
| CbpA | F | GGTTCTTACGGGCTAACATCAA | 516 | 57 |
|  | R | GACGCTCAATCTTAAACTGGACAC |  |  |
| FimA | F | CACTACGCTCACCATTCACAAG | 605 | 57 |
|  | R | GCTGTAATCCGCTTTGTCTGTG |  |  |
| FimC | F | TGTCGAAGGTGACGTTCTTCG | 843 | 57 |
|  | R | CAAGGTCACCGAGACTGCTGG |  |  |
| FimE | F | GCCCAGGACCGAGAGCGAGGGC | 775 | 57 |
|  | R | GCCTTCACAAATAACAGCAACC |  |  |
| FimG | F | ACGCTTCAGAAGGTCACCAGG | 929 | 57 |
|  | R | ATCTTGATCTGCCCCCATGCG |  |  |


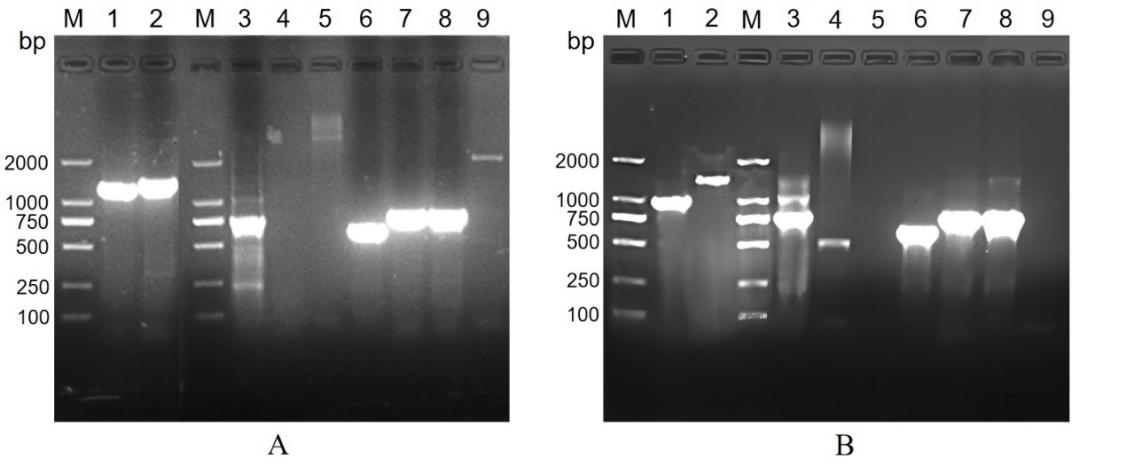


Supplementary Figure 2 S-*T. pyogenes* virulence gene detection results

M:DL2000 DNA Marker; 1:A:PLO-g1-1 gene, B:PLO-g1-2 gene; 2:PLO-g2 gene; 3:FimE gene; 4:CbpA gene; 5:FimG gene; 6:FimA gene; 7:NanH gene; 8:NanP gene; 9:FimC gene

The GenBank accession numbers for D-*T. pyogenes* and S-*T. pyogenes* are JARFUR000000000 and JBBVUN000000000, respectively. Eight primary virulence genes of S-*T. pyogenes*, including PLO, NanH, NanP, CbpA, FimA, FimC, FimE, and FimG, were detected by PCR. The results indicated that S-*T. pyogenes* harbored six virulence genes: PLO, FimA, FimE, NanH, NanP, and CbpA.

Supplementary Table 2 Pathogenicity test results of D-*T. pyogenes* in mice

| Dose (CFU/mL) | Total Mice (n) | Mortality (n) | Mortality (%) | LD_50_ |
| --- | --- | --- | --- | --- |
| 8.63×10^11^ | 6 | 6 | 100% | 6.8×10^10^ CFU |
| 7.55×10^11^ | 6 | 5 | 83.3% |  |
| 6.71×10^11^ | 6 | 4 | 66.7% |  |
| 5.49×10^11^ | 6 | 4 | 66.7% |  |
| 4.65×10^11^ | 6 | 3 | 50.0% |  |
| 4.03×10^11^ | 6 | 3 | 50.0% |  |
| 3.55×10^11^ | 6 | 4 | 66.7% |  |
| 3.02×10^11^ | 6 | 3 | 50.0% |  |
| 2.52×10^11^ | 6 | 3 | 50.0% |  |
| 2.16×10^11^ | 6 | 2 | 33.3% |  |
| 1.89×10^11^ | 6 | 2 | 33.3% |  |
| 1.68×10^11^ | 6 | 1 | 16.7% |  |
| Normal Saline | 6 | 0 | 0% |  |

Supplementary Table 3 Pathogenicity test results of S-*T. pyogenes* in mice

| Dose (CFU/mL) | Total Mice (n) | Mortality (n) | Mortality (%) | LD50 |
| --- | --- | --- | --- | --- |
| 6.0×10^8^ | 6 | 6 | 100% |  |
| 3.0×10^8^ | 6 | 6 | 100% |  |
| 1.5×10^8^ | 6 | 4 | 66.7% |  |
| 1.0×10^8^ | 6 | 3 | 50.0% | 2.3×10^7^ CFU |
| 7.5×10^7^ | 6 | 1 | 16.7% |  |
| 6.0×10^7^ | 6 | 0 | 0% |  |
| Normal Saline | 6 | 0 | 0% |  |

The median lethal dose (*LD₅₀*) was calculated using the modified Kärber method according to the following formula:

Log*LD_50_*​=log*D_max_*​−*d*×(∑*p*−0.5)

*D_max_*​: The maximum dose resulting in 100% mortality.

*d*: The log interval between adjacent dosage groups.

*p*: The mortality rate of each dose group (expressed as 0–1).

∑*p*: The sum of mortality rates starting from the 100% mortality group.

The number of mice in each dosage group was kept constant. Mortality rates were recorded for each inoculation dose, and the *LD_50_*​ was calculated based on the logarithmic dose intervals and cumulative mortality. A sample size of n = 6 per group was utilized. This sample size provides a minimum resolution of 16.7% and covers critical statistical increments such as 33.3%, 50.0%, 66.7%, and 83.3%. A sample size smaller than 6 may fail to capture an accurate 50% endpoint, while a sample size larger than 6 offers diminishing returns for *LD_50_*​ precision and conflicts with the 3R principles (Replacement, Reduction, and Refinement). Therefore, n = 6 provides a scientifically robust and ethically conservative calculation of the *LD_50_*​.

Bacterial DNA extraction methods：

Bacterial genomic DNA was extracted using the One-tube Clinical Sample DNA Extraction Kit (Sangon Biotech, Shanghai, China; Cat. No. B518403-0500). Briefly, purified single colonies were inoculated into 5 mL of Tryptic Soy Broth (TSB) supplemented with 5% fetal bovine serum (FBS) and incubated overnight at 37°C with shaking at 180 rpm. One milliliter of the bacterial culture was transferred to a 1.5 mL microcentrifuge tube and centrifuged at 12,000 rpm for 1 min. After discarding the supernatant, the bacterial pellet was resuspended in 100 µL of the extraction reagent. The suspension was boiled for 10 min to facilitate cell lysis and genomic DNA release. Following another centrifugation at 12,000 rpm for 1 min, the supernatant containing the template DNA was collected and stored for further analysis.

Cell lysis method:

The cell lysis buffer was prepared (50 mM KH_2_PO_4_, 300 mM KCl, and 1 mM EDTA), adjusted to pH 8.0, and sterilized by autoclaving. The sequence-verified recombinant plasmid pET32a-PLO was transformed into BL21 competent cells. The transformants were plated onto LB agar containing 100 μg/mL ampicillin (Amp) and incubated overnight at 37°C. Positive colonies were selected and expanded in 5 mL of LB broth supplemented with Amp.Subsequently, the culture was inoculated at a 1% ratio into 50 mL of LB broth (with Amp) and incubated in a constant temperature shaker at 37°C and 220 rpm until the OD_600_ reached 0.6–0.8. Protein expression was induced by adding IPTG to a final concentration of 1 mM, followed by incubation at 37°C and 180 rpm for 4 h. After induction, the bacterial cells were harvested by centrifugation at 8,000 rpm for 10 min at 4°C. The supernatant was discarded, and the cell pellet was resuspended in 5 mL of lysis buffer. The cells were then disrupted via ultrasonication in an ice bath (200 W, 10 min). The resulting lysate was centrifuged at 8,000 rpm for 10 min at 4°C to separate the soluble fraction (supernatant). The remaining pellet was resuspended in 5 mL of PBS to recover the insoluble protein fraction.

Kunming mice (approximately 20 g) used in this study were euthanized by a physical method of cervical dislocation, in accordance with the *AVMA Guidelines for the Euthanasia of Animals (2020 Edition)*.

The procedure was completed rapidly, and mice died as a result of respiratory and associated physiological failure. Briefly, the operator firmly grasped the tail near its base with the right hand and applied a rapid backward extension to align and stabilize the body, ensuring a swift and controlled movement to prevent struggling. The thumb and index finger of the left hand were positioned in a “V” shape and applied to the junction between the head and neck (the atlanto-occipital region, between the occipital bone and the first cervical vertebra) to accurately stabilize the cervical vertebrae. While maintaining tail extension, a rapid downward and backward force was applied with the left hand to dislocate the cervical vertebrae, particularly separating the first cervical vertebra from the occipital bone, thereby disrupting the connection between the spinal cord and brainstem. This step was completed within 1–2 seconds to ensure immediate loss of consciousness.

A slight clicking sound indicative of cervical dislocation, together with rapid relaxation of the limbs and cessation of respiration, was considered evidence that the procedure had been successfully performed. Loss of consciousness and death were confirmed by the absence of corneal reflexes, loss of voluntary movement, cessation of respiration, and complete muscle relaxation. If any involuntary movements were observed, suggesting incomplete dislocation, an immediate secondary physical method was applied to ensure death.
